# Effect of exposure parameters of cone beam computed tomography on metal artifact reduction around the dental implants in various bone densities

**DOI:** 10.1186/s12880-019-0334-4

**Published:** 2019-04-29

**Authors:** Abbas Shokri, Mohammad Reza Jamalpour, Atefeh Khavid, Zeinab Mohseni, Masoud Sadeghi

**Affiliations:** 1 Dental Implant Research Center, Department of Oral and Maxillofacial Radiology, Dental School, Hamadan University of Medical Sciences, Hamadan, Iran; 2 Dental Implant Research Center, Department of Oral and Maxillofacial Surgery, Dental School, Hamadan University of Medical Sciences, Hamadan, Iran; 3 Oral and Maxillofacial Radiology Department, Faculty of Dentistry, Kermanshah University of Medical Sciences, Kermanshah, Iran; 4 Medical Biology Research Center, Kermanshah University of Medical Sciences, Kermanshah, Iran

**Keywords:** Conebeam computed tomography, Dental implants, Metal artifacts, Bone density

## Abstract

**Background:**

This study aimed to assess the effect of exposure parameters such as milliampere (mA) and field of view (FOV) of cone beam computed tomography (CBCT) on a metal artifact of dental implants placed in different bone densities.

**Methods:**

A total of 27 bone blocks with different densities (nine were type 1, nine were types 2 and 3, and nine were type 4) were used in this in vitro, experimental study. These blocks were placed in mandibular wax models. The blocks were scanned after drilling (hole preparation) and after implant placement using Cranex3D imaging system with a 4 × 6 cm^2^and 6 × 8 cm^2^ FOV and 4 and 10 mA. Gray value of the bone blocks was recorded before and after placement of implants.

**Results:**

In general, irrespective of bone density, the amount of artifacts was lower in small FOV compared to large FOV (*P* < 0.05). Change of mA had no effect on metal artifacts (*P* > 0.05). Artifacts in type 4 bone were greater than in other bone types (*P* < 0.05). Difference between type 1 and types 2 and 3 was not significant (*P* > 0.05).

**Conclusion:**

According to the results of this study**,** Peri-implant artifacts were seen in all bone types; the amount of artifacts in type 4 bone was higher than that in other types. Size of FOV and bone density affect the metal artifacts around dental implants; so that a smaller FOV can be used to decrease metal artifacts.

## Background

Computed tomography (CT) is an important imaging modality for the detection of soft tissue lesions in the oral cavity and head and neck region [1]. However, Use of CT for dental purposes is limited due to high cost, large size of equipment and high patient radiation dose. Thus, cone beam computed tomography (CBCT) was introduced as an alternative diagnostic modality for dental applications [2]. It provides three-dimensional images for accurate diagnosis and treatment planning especially for dental implant placement [2–4]. In case of presence of metal in the scanning area, images are prone to artifacts [5]. The artifact is the main cause of reduction of image quality [6]. In some cases, metal artifacts make the image unusable. Some artifacts are created by a phenomenon known as beam hardening [6]. When X-ray beam passes through an object, photons with lower energy are absorbed compared to photons with higher energy. This phenomenon is known as beam hardening and is more commonly caused by high-density objects such as metal restorations and dental implants [6].

Considering the growing interest of dentists in use of CBCT as a standard diagnostic modality for different dental treatments such as dental implant placement, adequate knowledge about the amount of artifacts created with different exposure settings of CBCT in different bone densities is imperative [4–6].

In scanning of areas susceptible to beam hardening, decreasing the size of a field of view (FOV), changing patient position or separation of dental arches are recommended [6]. Exposure settings play an important role in this respect by affecting the energy of photons, and imaging with high voltage (kVp) has been recommended to decrease beam hardening [7]. The degree of rotation of device, shape of X-ray beams and type of algorithm used for data processing are among other factors that may play a role in beam hardening [7, 8]. Many previous studies have evaluated metal artifacts of CT and CBCT; however, most of them have been qualitative and only limited studies quantitatively evaluated metal artifacts [7]. Artifacts created around dental implants affect the clinical judgment of dentists. On the other hand, jawbone density is variable in patients and in different areas. Metal artifacts due to dental implants are variable in different bone densities. Thus, knowledge about the factors affecting the amount of metal artifacts and strategies to decrease them is important to improve image quality. Since image quality is an important factor in diagnosis and treatment planning.

### Aim

This study aimed to assess the in vitro effect of exposure parameters such as FOV and mA of CBCT system on the amounts of artifacts around dental implants in different bone densities.

## Methods

The study protocol was performed by in accordance with the Declaration of Helsinki and approved by the Ethics Committee of Hamadan University of Medical Sciences (ethical approval code: IR.UMSHA.REC.1395.519).

In this in vitro, experimental study, fresh bovine bone of a slaughtered cow with three different densities was used. According to the classification by Misch [9], D1 bone is comprised of completely cortical bone. This type of bone was obtained from the femur of a young cow. D2 and D3 bone types include both types of cortical and cancellous bones. This type of bone was obtained from a bovine rib. Its outer part was cortical and its internal part was cancellous bone. D4 bone type is comprised of cancellous bone. This type of bone was obtained from the internal part of a cow rib. Classification of bone types was done after their clinical examination.

First, bones were cut by a saw. Next, a milling machine was used to obtain cubes of bone with desired dimensions under water coolant. Cubic bone pieces had 90° angles, height and mesiodistal width of 10 mm and approximate thickness of 6.5 mm. Each implant was allocated an aseparate bone cube. For D2 and D3 bone types, samples had a layer of cortical bone in their buccal, lingual and upper surfaces with an internal cancellous bone at the center.

For each bone block, a buccal and a lingual surface were assigned and marked by a marker as B or L. A total of 27 bone pieces with 6.5 mm thickness were milled. Measurements were made by a caliper (Mitutoyo, Japan) with 0.02 mm accuracy. During the experiment, bone blocks were stored in a freezer to maintain their moisture.

**Fig. 1:**
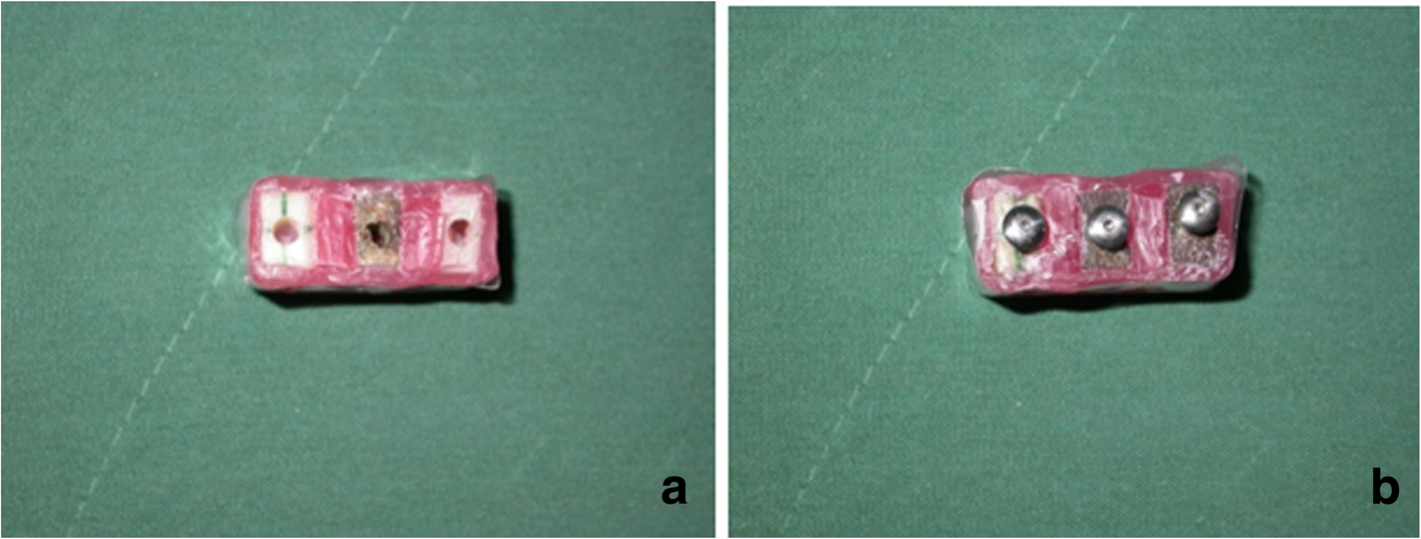
Bone blocks before (**a**) and after (**b**) insertion of implants in order to obtain three dimensional images

To mark the implant placement site, the center of the superior surface of each bone block was marked. To ensure accurate implant alignment, the center of the inferior surface of the bone block was also marked (Fig. 1).

Samples that were over-prepared or did not have the required thickness were excluded. Samples in which, implants were not inserted correctly were excluded.

The fixtures used were DIO (Centum seo-ro, Haeundae-gu, Busan, Korea), PSI type with a 3.5 mm diameter and 10 mm length, which were purchased from the manufacturer. Fixtures were placed in bone by a maxillofacial surgeon according to the instructions provided by the manufacturer.

**Fig. 2:**
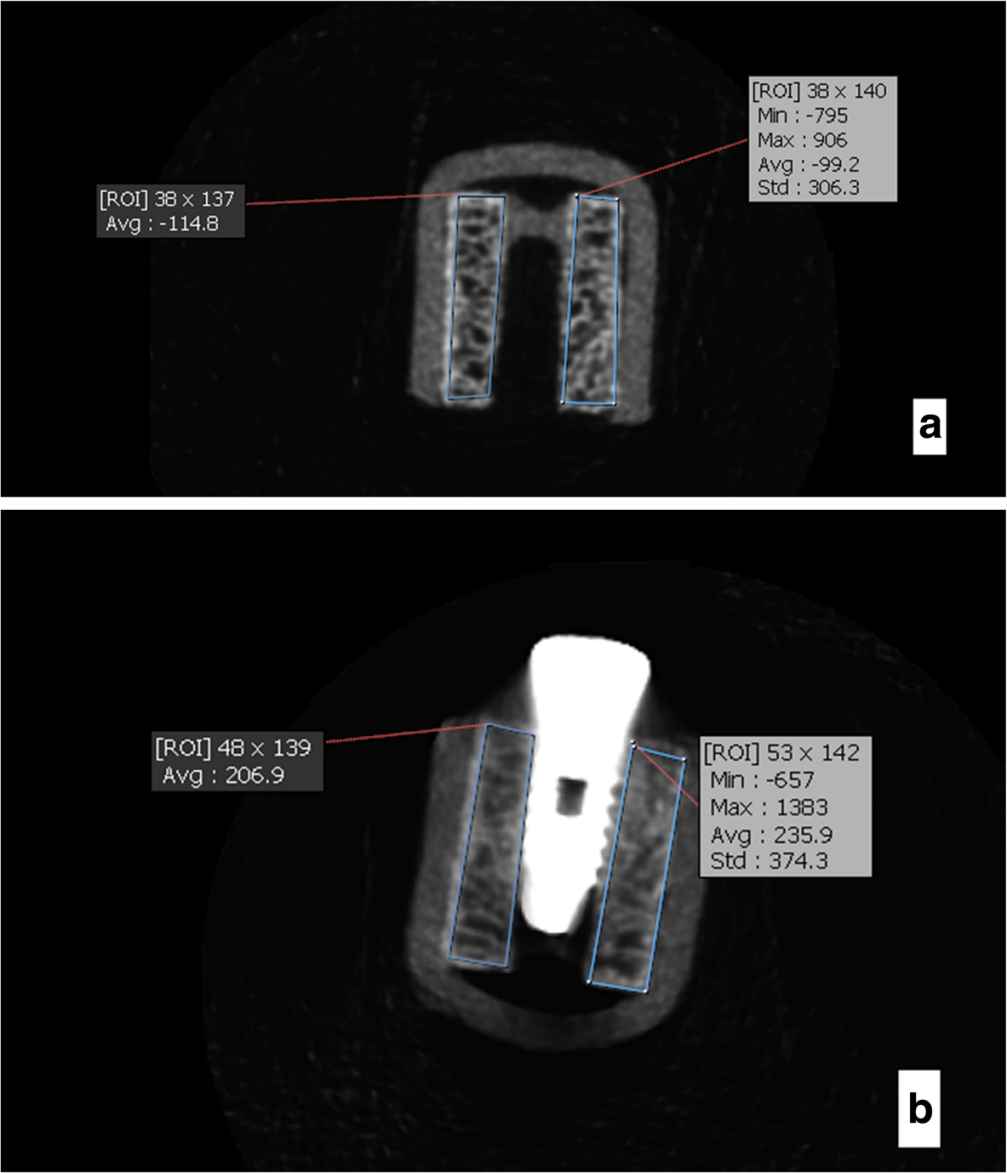
CBCT scans of the samples for types 2 and 3 bony density before (**a**) and after (**b**) insertion of implants in this exposure parameters: FOV: 6 × 8 cm^2^, mA: 4,T:12.6 s,kVp:90

**Fig. 3:**
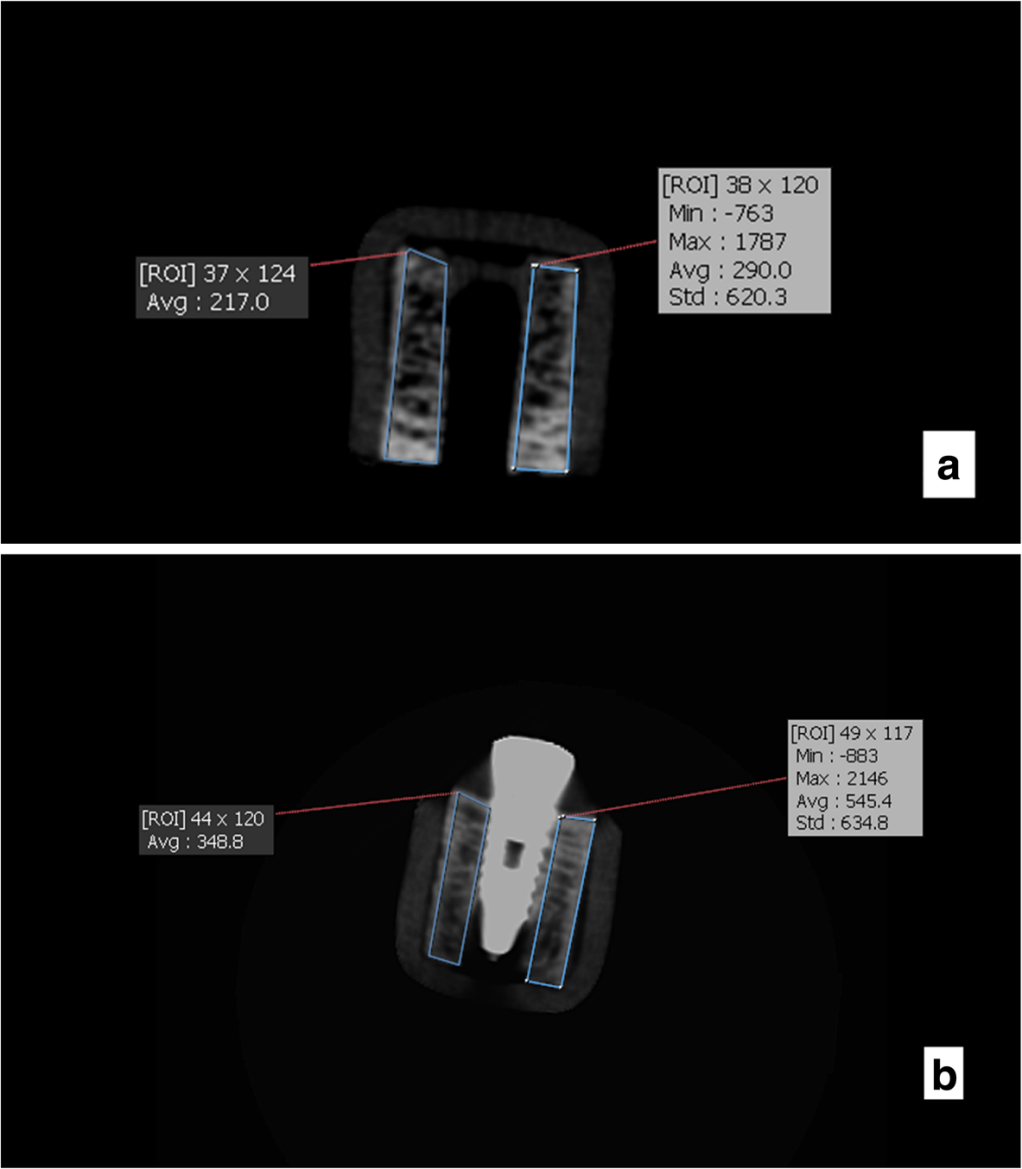
CBCT scans of the samples for types 2 and 3 bony density before (**a**) and after (**b**) insertion of implants in this exposure parameters: FOV: 6 × 8 cm^2^, mA: 10,T:12.6 s, kVp:90

**Fig. 4:**
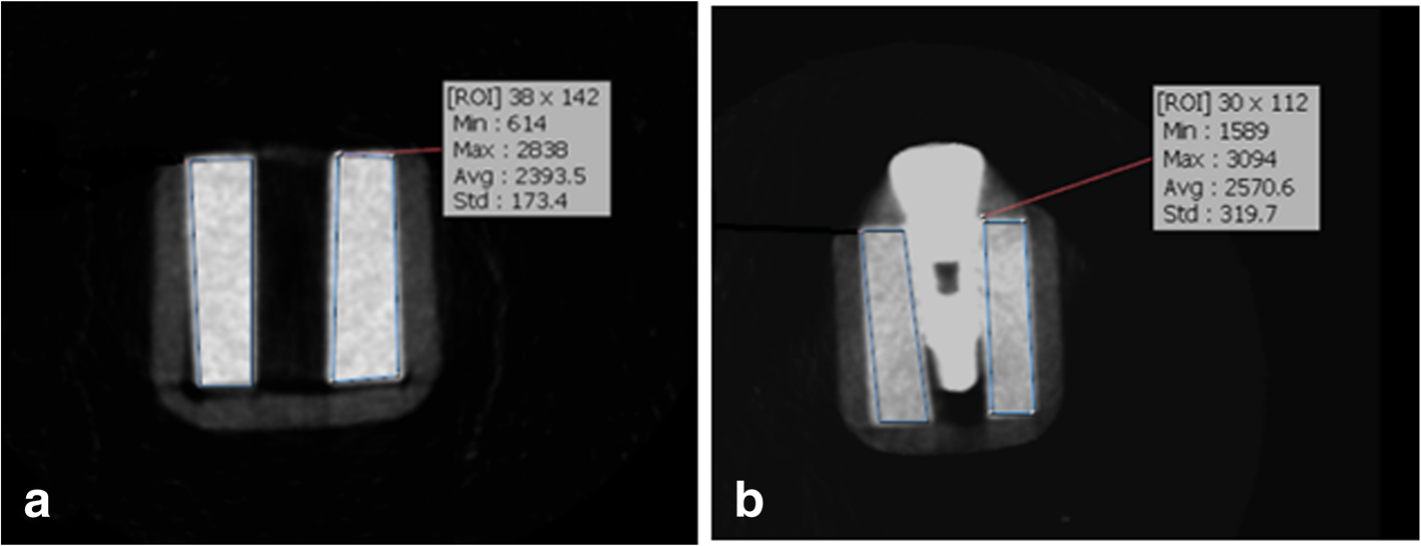
CBCT scans of the samples for type 1 bony density before (**a**) and after (**b**) insertion of implants in this exposure parameters: FOV: 4 × 6 cm^2^, mA: 4, T:6.1 s, kVp:90

**Fig. 5:**
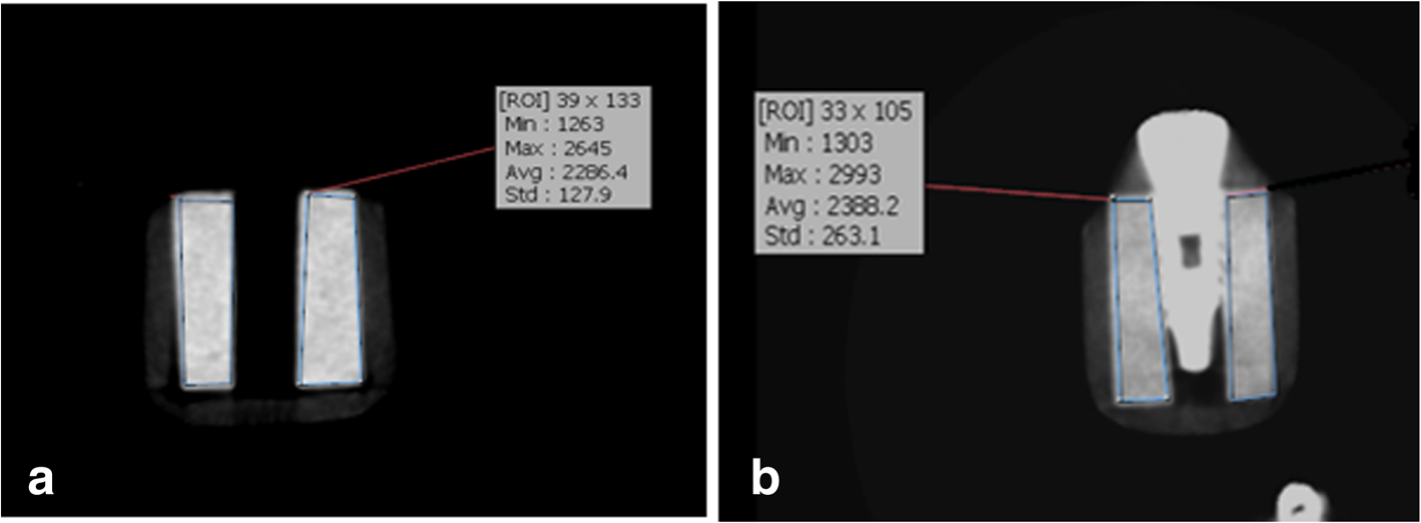
CBCT scans of the samples for type 1 bony density before (**a**) and after (**b**) insertion of implants in this exposure parameters: FOV: 4 × 6 cm^2^, mA: 10, T:6.1 s, kVp:90

**Fig. 6:**
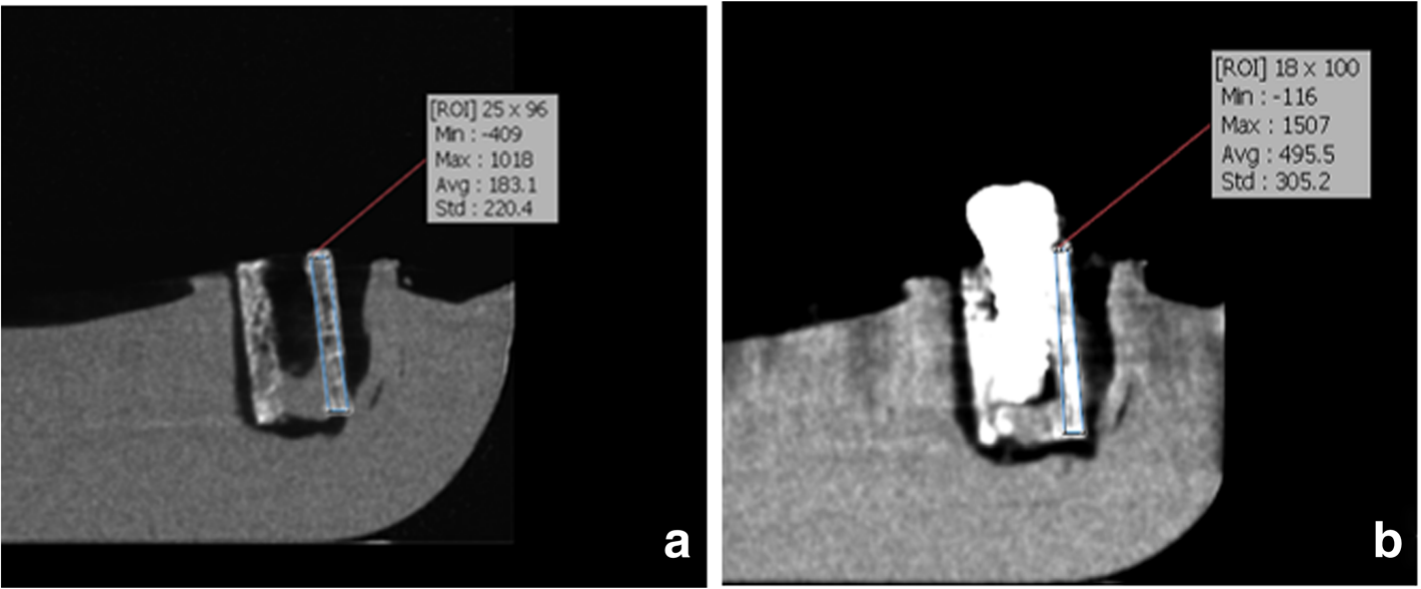
CBCT scans of the samples for type 4 bony density before (**a**) and after (**b**) insertion of implants in this exposure parameters: FOV: 6 × 8 cm^2^, mA: 4, T:12.6 s,kVp:90

**Fig. 7:**
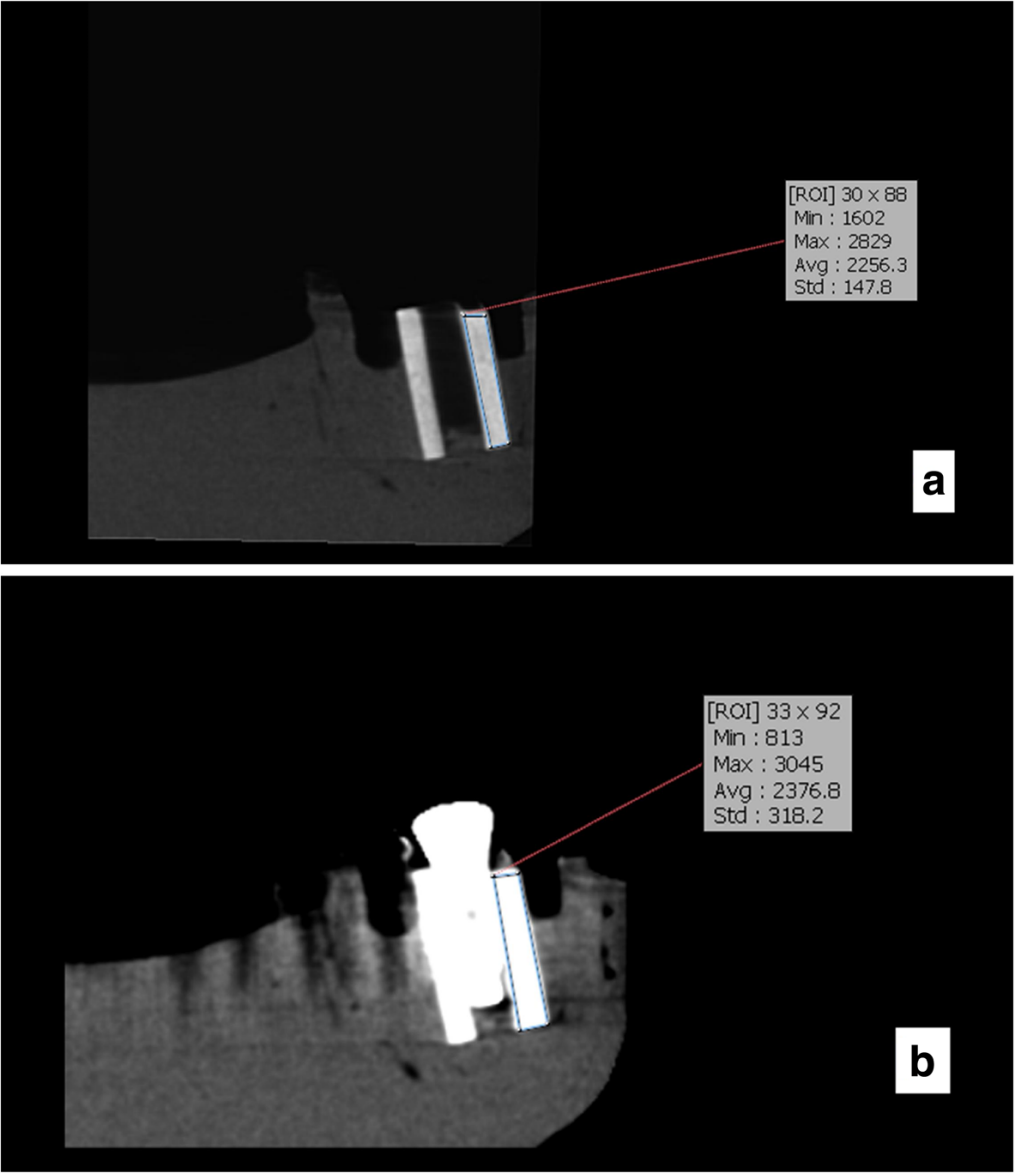
CBCT scans of the samples for type 4 bony density before (**a**) and after (**b**) insertion of implants in this exposure parameters: FOV: 6 × 8 cm^2^, mA: 10, T:12.6 s,kVp:90

After hole preparation and prior to placement of implants in bone, the bone blocks were coded and CBCT images were obtained to record the primary bone density prior to implant placement. Two layers of red wax were used for soft tissue simulation. Using a model of the mandible, wax layers were formed in the form of a mandible and nine implants were randomly placed in each mold and fixed. Mandibular wax model was only used for imaging with a large FOV. For imaging with a small FOV, a wax model of the mandible could not be used. Thus, samples were mounted in small wax models (three per each model); (Figs. 2, 3, 4, 5, 6 and 7).

The CBCT scans were obtained of samples with different exposure settings. The CBCT Cranex 3D (Soredex, Tuusula, Finland) with 90kVp and 0.2 mm voxel size for large FOV and 0.136 mm for small FOV were used in this study. For each bone type, four different exposure settings were used by CBCT imaging system:

1. FOV: 4 × 6 cm^2^ mA: 4 T:6.1 s kVp:90, Rotation time:10 s
2. FOV: 4 × 6cm^2^mA: 10 T:6.1 s kVp:90, Rotation time:10 s
3. FOV: 6 × 8cm^2^mA: 4 T:12.6 s kVp:90, Rotation time:20 s
4. FOV: 6 × 8cm^2^mA: 10 T:12.6 s kVp:90, Rotation time:20 s

The mean gray value in the region of interest (implant site) was obtained in the buccal surface of bone blocks.

Next, implants were placed in bone blocks and CBCT scans were obtained again with the same conditions that mentioned above.

Before placement of implants, marking the upper and lower levels of the bony blocks, the central area of the block was determined, and the path of the implant drill and insertion was precisely placed in the middle of the bone.

In cbct images, with cross-section sections displaced, exactly the middle area of each bone was used to determine the mean gray value. By doing so, the areas selected were completely identical before and after implant placement.

Bone density in the region of interest was determined (Figs. 2, 3, 4, 5, 6 and 7). Based on the mean change in a gray value of these areas, the amount of metal artifacts of dental implants in different bone types due to change in exposure settings was evaluated.

The amount of artifacts caused by dental implants was calculated by subtracting the mean gray value before and after implant placement, which was similar to the method used by Benic et al. [10] for artifact calculation.

For the scanning, the study models were positioned on the supporting plate provided by the manufacturer with the occlusal plane parallel to the horizontal plane and thereafter positioned in the center of the field of view (FOV) using the laser orientation beams. The CBCT scans were obtained.

Axial reconstructions perpendicular to the implant’s longitudinal axis were used for the data evaluation. The dimensions of the region of interest (ROI) were set at 0.25 mm 9 0.25 mm in the axial plane a 4 mm in the implant longitudinal axis, resulting in a total ROI of 16 voxels. Along the implant axis, ROI extended from 3 to 7 mm apically to the implant shoulder. Xray attenuation expressed as gray value (GV) was recorded in buccal surface of bone blocks.

To facilitate the reproducibility of the measurement, a transparent acetate foil with the printed implant and ROI outlines was placed over the CBCT images on the computer monitor. The software provided one mean GV for each ROI. Subsequently, cone-beam computed tomography images of the control models without implants were assessed. Anatomical landmarks on the model’s surface (e.g. teeth and alveolar ridge) were used to identify volumes corresponding to those containing implants in the test models. These volumes were labeled using the previously described acetate foil with printed implant outline. Thereafter, GV were measured in the ROI corresponding to those assessed in the test models with implants [10].

All CBCT scans were reconstructed by OnDemand 3D Dental software (Cybermed, Seoul, Korea) and analyzed. The primary traced arch for each sample was modified such that a precise image was obtained for each implant and cross-sections passed right through the center of each bone block perpendicular to them.

Considering that ROI was selected to determine the mean gray value after the preparation of the implant cavity, the remaining bone around the cavity was completely selected in the upper and lower directions as well as buccolingual to determine the ROI. By choosing the central cut after placement of the implant, again, the area was selected completely around the implant and the mean gray value was determined.

All scans were evaluated by two oral and maxillofacial radiologists twice with a 2-week interval. To assess the intraobserver and inter-observer agreements, the intra- and inter-class correlation coefficients (ICC) were calculated. Both quantitative and qualitative analyzes were used to in this study. Normality of the data was assessed using the Kolmogorov-Smirnov test. Repeated measures ANOVA and LSD test were then applied for data analysis.

## Results

The interclass correlation coefficient was 93% and the intra-class correlation coefficient was 97% in our study. These values indicated high (excellent) inter- and intra-examiner reliability.

The mean, standard deviation, minimum and maximum values of artifacts were calculated. Table 1 shows the amount of artifacts in different exposure settings. As shown in Table 1, the highest mean artifact around dental implants was 438.07 in use of large FOV and 4 mA while the lowest mean artifact was 266.88 and obtained by using a small FOV and 10 mA exposure settings.

**Table 1:** Amount of artifacts in different exposure settings (*n* = 27)

Amount of artifact	Mean	Standard deviation
Small FOV, 4 mA	295.19	111.68547
Small FOV, 10 mA	266.88	124.79808
Large FOV, 4 mA	438.07	261.79284
Large FOV, 10 mA	424.78	302.87518

Table 2 shows the amount of artifacts in different bone types. As shown in Table 2, the highest mean amount of artifacts was noted in type 4 bone-in uses of large FOV and 4 mA while the lowest mean amount of artifacts was noted in type 1 bone with small FOV and 10 mA.

**Table 2:** Amount of artifacts in different bone types (*n* = 27)

Bone type	Exposure settings	Mean	Standard deviation
Type 1	Small FOV, 4 mA	209.83	61.23145
	Small FOV, 10 mA	174.11	89.35054
	Large FOV, 4 mA	382.98	239.04508
	Large FOV, 10 mA	342.97	229.02314
Types 2 and 3	Small FOV, 4 mA	263.53	77.44921
	Small FOV, 10 mA	283.42	101.28344
	Large FOV, 4 mA	360.98	224.80996
	Large FOV, 10 mA	382.60	223.14333
Type 4	Small FOV, 4 mA	412.19	77.89704
	Small FOV, 10 mA	343.11	125.8897
	Large FOV, 4 mA	570.24	292.o681
	Large FOV, 10 mA	548.77	411.1727

Table 3 shows the results of the Kolmogorov-Smirnov test regarding the normality of the data. As shown in Table 3, the data had the normal distribution in all bone types and exposure settings. Repeated measures ANOVA was then applied and LSD test was used for pairwise comparisons.

**Table 3:** Results of Kolmogorov-Smirnov test regarding the normality of the data

Bone type	Settings	Coefficient	*P* value
Type 1	Small FOV, 4 mA	0.622	0.834
	Small FOV, 10 mA	0.571	0.900
	Large FOV, 4 mA	0.493	0.968
	Large FOV, 10 mA	0.669	0.761
Types 2 and 3	Small FOV, 4 mA	0.661	0.775
	Small FOV, 10 mA	0.538	0.935
	Large FOV, 4 mA	0.601	0.863
	Large FOV, 10 mA	0.446	0.988
Type 4	Small FOV, 4 mA	0.516	0.952
	Small FOV, 10 mA	0.554	0.929
	Large FOV, 4 mA	0.670	0.745
	Large FOV, 10 mA	0.717	0.682

Considering the presence of a significant difference, pairwise comparisons were performed by LSD test. Table 4 shows the results of LSD test for pairwise comparisons. According to this test, the amount of artifacts in small FOV (4 × 6 cm^2^) and 4 mA was significantly less than that in large FOV (6 × 8 cm^2^) with 4 mA and large FOV with 10 mA (*P* < 0.05). Also, the amount of artifacts in small FOV (4 × 6 cm^2^) and 10 mA was significantly less than that in large FOV and 10 mA and large FOV (6 × 8 cm^2^) and 4 mA (*P* < 0.05).

**Table 4:** Results of LSD test for pairwise comparisons of the combinations of exposure settings

Artifact	Artifact	Subtraction of means	Standard error	P value
Small FOV, 4 mA	Small FOV, 10 mA	28.304	23.132	0.232
	Large FOV, 4 mA	− 142.881	48.753	0.007
	Large FOV, 10 mA	− 129.593	56.036	0.029
Small FOV, 10 mA	Small FOV, 4 mA	−28.304	23.132	0.232
	Large FOV, 4 mA	−171.185	48.504	0.002
	Large FOV, 10 mA	− 157.896	57.415	0.011
Large FOV, 4 mA	Small FOV, 4 mA	142.881	48.753	0.007
	Small FOV, 10 mA	171.185	48.504	0.002
	Large FOV, 10 mA	13.289	40.006	0.742
Large FOV, 10 mA	Small FOV, 4 mA	129.593	56.036	0.029
	Small FOV, 10 mA	157.896	57.415	0.011
	Large FOV, 4 mA	−13.289	40.006	0.742

Comparison of different bone types in terms of the amount of artifacts by repeated measures ANOVA showed a significant difference among different bone types (*P* = 0.019). Thus, LSD test was applied for pairwise comparisons of bone types; the results are shown in Table 5.

**Table 5:** Pairwise comparison of different bone types using LSD test

Density	Density	Mean of subtraction	Standard error	P value
1	2, 3	−45.1611	65.32473	0.496
	4	− 191.1056	65.32473	0.007
2, 3	1	45.1611	65.32473	0.496
	4	−145.9444	65.32473	0.035
4	1	191.1056	65.32473	0.007
	2, 3	145.9444	65.32473	0.035

Table 5 shows that type 4 bone had significantly higher amount of artifacts than types 1, and 2 and 3 (*P* < 0.05).

## Discussion

Considering the increasing use of CBCT by dentists as a standard diagnostic tool for different treatments such as implant placement, knowledge about the effect of exposure settings on the amount of artifacts around dental implants seems imperative [11, 12].

Beam hardening decreases the quality of the image and some light streaks reflected from the metal object or completely dark areas adjacent to the metal object may be seen. Artifacts created around dental implants may affect the dentists’ clinical judgment [13–15].

The projection modification method has been increasingly favored in recent years because of its simplicity. In the projection modification approach, the metal shadows in the raw projection data caused by the X-ray passing through the metallic implants are first segmented and then replaced using some estimated values.

Jawbone density is variable in patients in different parts of the jaw and undoubtedly, the effect of metal artifacts of dental implants would be different in different bone densities. This study was carried out to assess the effect of factors affecting the amount of metal artifacts and to find strategies to decrease them and improve the quality of image.

Various parameters such as device type, voltage (KVP), field of view (FOV), milliampere (mA) and time can affect the amount of metal artifact created by the dental implant in the CBCT images. The effect of time and mA on creating metal artifacts in the CBCT images is similar. On the other hand, to minimize artifacts from patient movement (motion blurs), time should be set at the minimum; for this reason, only the effect of mA has been studied in this study. Also, in all devices, the voltage is set to 90 to 95 kV and is constant; this is due to the minimization of scattered radiation.

Also, on all devices, the field of view is selected based on the area requested by the doctor and mA based on the patient’s size. Because this study aimed to assess the effect of exposure parameters such as milliampere (mA) and field of view (FOV) of cone beam computed tomography (CBCT) on metal artifact of dental implants placed in different bone densities.

Comparison of FOVs with the same amperage showed that artifacts were less around dental implants in small FOV, which may be due to the fact that by an increase in FOV, the irradiated area increases in size and consequently, scattered radiation, noise and image artifact increase. This finding was in line with that of Parsa et al. [16]. They showed that in Accuitomo X-ray unit, gray value and artifact increased at the implant site by increasing the FOV. The similarity between our results and those of Parsa et al. [16] may be due to the similarity of exposure parameters such as voltage (kVp) and amperage (mA) in Accuitomo and Soredex units. In Accuitomo, exposure settings were 90kVp and 5 mA while we used 4 and 10 mA and 90kVp. On the other hand, our findings were not in line with those of Parsa et al., [16] who used NewTom CBCT unit since they reported that by an increase in FOV, gray value and artifact at the implant site decreased, which may be due to differences in exposure parameters such as voltage and amperage and also difference in image reconstruction and post-processing methods. It should be noted that the detector of NewTom images IIT/CCD while in our study, flat panel detector was used. The flat panel detector has higher contrast and spatial resolution than IIT/CCD. On the other hand, artifact and pixel noise of IIT/CCD are higher than those of flat panel detectors [17]. Thus, the difference in detectors used can be another reason for different results obtained by Parsa et al. [16], in use of the NewTom unit compared to ours.

Size and amount of FOVs are different in different models of CBCT units by different manufacturers [18]. Size of the selected FOV is the most important scanning factor limiting the patient radiation dose and determining the quality of the image [16]. Our results indicated that this factor affected the amount of artifacts around dental implants, the amount of artifacts in small FOV (4 × 6 cm^2^) and 4 mA was significantly less than that in large FOV (6 × 8 cm^2^) with 4 mA and large FOV with 10 mA (*P* < 0.05). Also, the amount of artifacts in small FOV (4 × 6 cm^2^) and 10 mA was significantly less than that in large FOV and 10 mA and large FOV (6 × 8 cm^2^) and 4 mA (*P* < 0.05). In our study, comparison of amperage (4 and 10 mA) in the same FOV showed that although the amount of artifact in small FOV and 4 mA was less than that in small FOV and 10 mA, and the amount of artifact in large FOV and 10 mA was less than that in large FOV and 4 mA, these differences were not statistically significant. It may be concluded that mA does not affect the amount of artifacts around dental implants.

Chindasombatjareon et al. [19] found similar results and showed that amperage had no significant effect on metal artifacts. They used MDCT and CBCT while we used CBCT. Moreover, we only used titanium implants while they used titanium, aluminum, cobalt, and chrome and type IV gold alloy cubes. Their superiority to our study was that they evaluated the artifacts of most metals commonly used in dentistry but their findings confirmed our results.

Our findings regarding no significant effect of amperage on metal artifacts were in agreement with the results of Pauwelse et al. [13]. In their study, despite the effect of amperage on metal artifacts around titanium and lead rods in 10–180-degree range and in 13 different CBCT units, no difference was found in metal artifacts between high amperage (mA) and low amperage modes. Even with a 88% increase in mA, no difference occurred in metal artifacts.

Among exposure parameters (amperage, time and voltage), voltage (kVp) appears to be the most important factor affecting metal artifacts around dental implants. Chindasombatjareon et al. [19] showed that the amount of metal artifacts decreased by an increase in voltage (kVp). The results of Esmaelli et al., [7] also confirmed that increasing the voltage (kVp) can decrease metal artifacts. They compared NewTom and Planmeca CBCT and MSCT and found that increasing the voltage (kVp) decreased the artifacts and justified this finding by the effect of voltage on the energy of photons. Schulze et al., [3] also confirmed the effect of voltage (kVp) on metal artifacts and stated that a significant difference existed between high- and low-energy beams in imaging of high-density objects.

In our study, the assessment of bone types revealed that the amount of artifacts had a significant difference among different bone types (*P* = 0.019) and type 4 bone had significantly higher amount of artifacts than types 1, and 2 and 3 (*P* < 0.05).

Type 1 bone includes homogenous compact bone; type 2 bone includes thick cortical bone along with bone marrow spaces; type 3 includes thin cortical bone along with dense trabecular bone with good strength and type 4 includes thin cortical bone with low density and low strength bone.

In general, the higher the bone density is, the lower is the amount of photons passed through the bone and consequently, the amount of photons reaching the implant decreases. Consequently, X-ray beam scattering would be less. On the other hand, by absorbing low-energy photons by high-density bone and passage of high-energy photons, the mean energy of photons increases, which exerts the same effect as increasing the voltage (kVp) on the region of interest. Considering the results of Chindasombatjareon et al., [19] Esmaelli et al., [7] and Schulze et al., [3] on the effect of increasing the voltage (kVp) on metal artifacts, it seems that higher bone density decreases the artifacts around dental implants.

Thus, type 4 bone, with the lowest amount of cortical bone density has the least beam attenuation. High energy photons in high amounts impact metal structures such as implant and result in high X-ray beam scattering and eventually higher image artifact in this type of bone.

One of the most important strengths of our study compared to others is the use of bones with different densities in a mandibular wax model while a gypsum model [10, 20–22] or poly-methyl methacrylate [13] were used in previous studies. It has been stated that use of phantom due to homogeneity has equal gray scale value in all points. However, in the clinical setting, jawbones are heterogeneous, affecting the gray value measurements [13]. Thus, our study conditions were closer to the clinical setting.

A previous study used a coding system by the observers to assess the amount of metal artifacts. Code 5 indicated no metal artifact; codes 4, 3 and 2 indicated that over 90, 75 and 50% of implant structure was well visualized. Code 1 was allocated when less than 50% of the implant structure was well visualized [7]. However, in our study, the amount of gray value was precisely measured before and after implant placement to assess the effect of different factors on metal artifacts. Also, in our study, region of interest was determined with high precision and we tried our best to match the test and control groups while in a previous study [3], determination of region of interest was not explained in detail or in another study, assessment was only done after implant insertion [8].

Currently, Numerous methods and algorithms have been proposed to reduce metal artifacts in CT and CBCT images [23–26]. Y. Chen et al., [23] showed that A discriminative dictionary representation method was developed to mitigate CT truncation artifacts directly in the DICOM image domain. Both phantom and human subject studies demonstrated that the proposed method can effectively reduce truncation artifacts without access to projection data. Liu J, et al. [26] used a 3D feature constrained reconstruction (3D-FCR) algorithm for Low-dose computed tomography (LDCT) image reconstruction. Qualitative and quantitative results show that the proposed method can lead to a promising improvement of LDCT image quality. Candemil AP, et al. [25] evaluated the effectiveness of metal artefact reduction (MAR) in cone beam computed tomography (CBCT) artefacts arising from metallic objects in the exomass. Results showed MAR increased significantly mean voxel values (*p* ≤ 0.05) in the presence of titanium, and presented no significant difference (*p* > 0.05) for CoCr. Voxel value variability did not differ significantly (*p* > 0.05) for both materials. In their conclusion, MAR was not effective to correct CBCT artefacts arising from metallic objects in the exomass in the two CBCT units used.

In this study, these methods and algorithms are not used to reduce artifacts of images. It is suggested that in later studies, their effectiveness should be considered in order to reduce artifacts of CBCT images.

Our study had some limitations as well. We used bone blocks fixed in wax and paraffin and scanned them while for better simulation of clinical setting; an actual mandible had to be used.

Our results highlighted the fact that artifacts always exist around dental implants. Thus, clinicians must be careful in the interpretation of these images. In contrast to the general belief of many clinicians that changing the amperage can affect the amount of artifacts, our results showed that bone density was the main factor determining the effect of exposure parameters on artifacts.

## Conclusions

According to the results of this study**,** Peri-implant artifacts were seen in all bone types; the amount of artifacts in type 4 bone was higher than that in other types. Size of FOV and bone density affect the metal artifacts around dental implants; so that a smaller FOV can be used to decrease metal artifacts. In addition, the increasing the amperage (mA) does not decrease the metal artifacts.

## Abbreviations

CBCT: Cone beam computed tomography
CT: Computed tomography
FOV: Field of view
mA: Milliampere
